# Unique Spin Vortices and Topological Charges in Quantum Dots with Spin-orbit Couplings

**DOI:** 10.1038/s41598-018-35837-y

**Published:** 2019-01-24

**Authors:** Wenchen Luo, Amin Naseri, Jesko Sirker, Tapash Chakraborty

**Affiliations:** 1 Department of Physics, School of Physics and Electronics, Central South University, Changsha, Hunan 410083 P. R. China; 2 Department of Physics and Astronomy, University of Manitoba, Winnipeg, R3T 2N2 Canada

## Abstract

Spin textures of one or two electrons in a quantum dot with Rashba or Dresselhaus spin-orbit couplings reveal several intriguing properties. We show here that even at the single-electron level *stable* spin vortices with *tunable* topological charges exist. These topological textures appear in the *ground state* of the dots. The textures are stabilized by time-reversal symmetry breaking and are robust against the eccentricity of the dot. The topological charge is directly related to the sign of the *z* component of the spin in a large dot, allowing a direct probe of its topological properties. This would clearly pave the way to possible future topological spintronics. The phenomenon of spin vortices persists for the interacting two-electron dot in the presence of a magnetic field.

## Introduction

A variety of topological states have recently been observed in condensed matter physics. These novel states of matter are a direct consequence of spin-orbit coupling (SOC)^1,2^ with topological insulators (TIs) being one of the most prominent examples^3,4^. The SOC also plays an important role in tailoring topological superconductors (TSs) where the elusive Majorana fermions might be present^5,6^. Both TIs and TSs display a topologically non-trivial structure in momentum space. SOC can, however, also lead to topological charges in real space. The Dzyaloshinskii-Moriya interaction —microscopically based on the SOC—can, for example, give rise to spin skyrmions in helical magnets^7,8^ and pseudospin skyrmions in bilayer graphene^9,10^. Synthetic spin-orbit couplings can also be engineered in cold atomic gases and skyrmion-like spin textures^11,12^ have been observed. For pseudospinor condensates, the existence of vortex solitons that are stabilized by the combined Rashba and Dresselhaus SOC has been predicted^13,14^.

Quantum dots (QDs) are of practical and fundamental interest and provide an excellent platform to control the spin and charge of a single electron^15–17^. Extensive studies on QDs with SOCs have been reported in recent years^18–33^. Here we investigate the spin textures associated with the electron density profiles in isotropic and elliptical QDs. We show that in the presence of SOC the in-plane spin texture of a single electron is a spin vortex. The QD is consequently turned into an artificial atom^34^ with topological features. Spin vortices often emerge in many-spin systems forming either a crystalline arrangement or vortex/anti-vortex pairs^35,36^. For instance, in quantum Hall systems the skyrmion is a single-particle excitation in low Landau levels and the in-plane spin texture is similar to the one we find in a QD with SOC. The skyrmion excitations in the former case are, however, induced by Coulomb interactions. Interaction-induced merons have also been described for rotationally symmetric QD’s at specific magnetic fields where states with different angular momentum cross^37^. The rotational symmetry of the dot and the conservation of total angular momentum are also at the heart of the meron-like spin textures observed in cylindrical dots with a large *z* component thickness in the presence of linear and cubic Dresselhaus SOCs^38^. In contrast, the spin textures described in the following are stable and tunable, are neither skyrmions nor merons, and exist even without the rotational symmetry, at the single- and multi-electron level, and for any given magnetic field.

We focus on the physics of the two-dimensional (2D) surface where the QD is constructed. We consider both the Rashba and the linear Dresselhaus SOCs which arise in materials with broken inversion symmetry. The strength of the Rashba SOC can be controlled by a gate electric field^39–41^. Moreover, the ratio of the Rashba SOC to the Dresselhaus SOC can be tuned over a wide range, for instance in InAs QDs, by applying an in-plane magnetic field^42^. We will show that this leads to a system where the topological charge can be dynamically controlled by external electromagnetic fields making spin vortices in QDs possible candidates for future applications in topological spintronics and quantum information.

## Results

The SOCs can be theoretically considered as effective momentum-dependent magnetic fields^43^. In the absence of a confinement and an external magnetic field, the momentum is conserved and the SOC in the Hamiltonian becomes a momentum-dependent operator with a good quantum number (e.g., the helicity operator for Rashba SOC). On the other hand, the spin state is momentum-independent if both Rashba and Dresselhaus couplings have equal strength and there is no Zeeman coupling, leading to a persistent spin helix^44,45^. This particular spin state persists in the presence of a confinement potential and can be obtained by exactly solving the Hamiltonian which is equivalent to a quantum Rabi model (See the supplementary material for details). If the spin is not a good quantum number then it is instructive to study the spin field in a given single-particle wavefunction Ψ(**r**) of the dot

1 $${\sigma }_{i}({\bf{r}})={{\rm{\Psi }}}^{\dagger }({\bf{r}}){\sigma }_{i}{\rm{\Psi }}({\bf{r}}),$$

where *σ*_*i*_ for *i* = *x*, *y*, *z* are Pauli matrices. An in-plane vector field ${\boldsymbol{\sigma }}({\bf{r}})=({\sigma }_{x}({\bf{r}}),{\sigma }_{y}({\bf{r}}))$ reveals how the spin in real space is locally affected by the effective magnetic field. In the following, we demonstrate that generic SOCs compel the spin field to rotate around the center of the QD and to develop into a spin vortex.

### Model

The Hamiltonian of an electron with effective mass m* and charge −*e* in a quantum dot with SOCs is given by

2 $$H=\frac{{({\bf{p}}+e{\bf{A}})}^{2}}{2{m}^{\ast }}+\frac{{m}^{\ast }}{2}({\omega }_{x}^{2}{x}^{2}+{\omega }_{y}^{2}{y}^{2})+\frac{{\rm{\Delta }}{\sigma }_{z}}{2}+{H}_{SOC},$$

where the vector potential is chosen in the symmetric gauge ${\bf{A}}=\frac{1}{2}B\,(\,-\,y,x,0)$ with the magnetic field *B*. The confinement is anisotropic with the frequencies in two directions, *ω*_*x*_ and *ω*_*y*_, and Δ is the Zeeman coupling. We consider both the Rashba SOC, *H*_*R*_, and the Dresselhaus SOC, *H*_*D*_, with

3 $${H}_{R}={g}_{1}\,({\sigma }_{x}{P}_{y}-{\sigma }_{y}{P}_{x}),$$

4 $${H}_{D}={g}_{2}\,({\sigma }_{y}{P}_{y}-{\sigma }_{x}{P}_{x}),$$

where *H*_*SOC*_ = *H*_*R*_ + *H*_*D*_. *P*_*i*_ = *p*_*i*_ + *eA*_*i*_ is the kinetic momentum, and *g*_1,2_ determine the strength of each SOC. We note that Rashba and Dresselhaus terms have different rotational symmetry generators: *H*_*R*_ commutes with ${L}_{z}+\hslash {\sigma }_{z}/2$ while *H*_*D*_ commutes with ${L}_{z}-\hslash {\sigma }_{z}/2$, where *L*_*z*_ is the *z*-component of the angular momentum operator. In the following, we will show that this difference is responsible for the different topological charges associated with the spin vortex of the dot.

It is also useful to introduce a renormalized set of frequencies ${{\rm{\Omega }}}_{i}=\sqrt{{\omega }_{i}^{2}+{\omega }_{c}^{2}/4}$ with the cyclotron frequency *ω*_*c*_ = *eB*/m*. The natural length scales in *x* and *y* directions are ${\ell }_{i}=\sqrt{\hslash /({m}^{\ast }{{\rm{\Omega }}}_{i})}$ while the confinement lengths are defined as ${R}_{i}=\sqrt{\hslash /({m}^{\ast }{\omega }_{i})}$. In the numerical calculations presented in the following the eigenvectors of ${H}_{0}=\frac{{{\bf{p}}}^{2}}{2{m}^{\ast }}+\frac{{m}^{\ast }}{2}({{\rm{\Omega }}}_{x}^{2}{x}^{2}+{{\rm{\Omega }}}_{y}^{2}{y}^{2})+\frac{{\rm{\Delta }}}{2}{\sigma }_{z}$, which is a two-dimensional harmonic oscillator, are used as a basis set. To be concrete, we consider the case of an InAs dot here, where the effective mass is m* = 0.042*m*_*e*_, Landé factor *g*_*L*_ = −14 and dielectric constant $\epsilon =14.6$. In this system it appears to be experimentally feasible to change the ratio of the SOCs *g*_1_/*g*_2_ over a wide range.

### Exact and perturbative calculations

**Figure 1:**
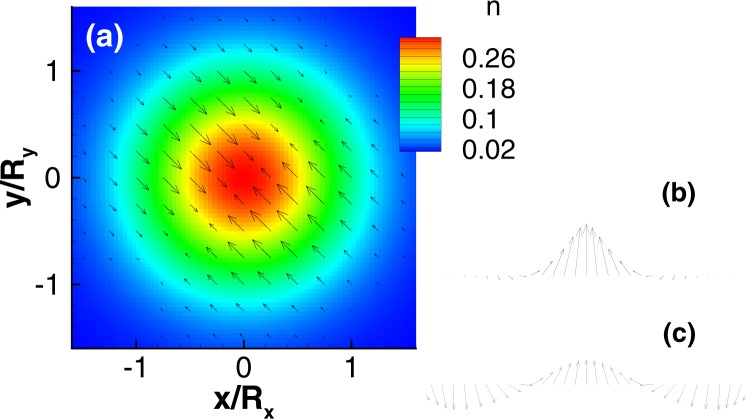
Numerical results for a single-electron QD with *R*_*x*_ = *R*_*y*_ = 35 nm, *B* = 0.1 T, and equal SOCs $\hslash {g}_{1}=\hslash {g}_{2}=20$ nm · meV. (**a**) Electron density (contours) and in-plane spin fields (arrows), (**b**) *σ*_*z*_(**r**) along *x* = −*y*, and (**c**) the normalized ${\tilde{\sigma }}_{z}({\bf{r}})={\sigma }_{z}({\bf{r}})/\sqrt{{\boldsymbol{\sigma }}{({\bf{r}})}^{2}+{\sigma }_{z}{({\bf{r}})}^{2}}$ along *x* = −*y*.

No analytical solution is known for the generic Hamiltonian in Eq. (2) due to its complexity^46^. We can, however, analytically investigate the special case of an isotropic dot (Ω_*x*,*y*_ = Ω, ${\ell }_{x,y}=\ell$) without a magnetic field and with equal SOCs, *g*_1,2_ = *g*. The Hamiltonian (2) is then equivalent to a two-component quantum Rabi model which has been extensively studied in quantum optics (See the supplementary material for details). The ground states in this case are a degenerate Kramers pair due to time reversal symmetry,

5 $$|GS{\rangle }_{\pm }=\frac{1}{\sqrt{2}}{e}^{\pm i\sqrt{2}{m}^{\ast }(y-x)g/\hslash }(\begin{array}{c}\pm {e}^{-i\pi /4}\\ 1\end{array})\,|0,0\rangle $$

where $|0,0\rangle$ is the ground state of the two-dimensional quantum oscillator *H*_0_. A weak magnetic field will lift the degeneracy of the Kramers pair, and the unique ground state is then given by $|GS\rangle ={(|GS\rangle }_{+}+{\rm{sgn}}({\rm{\Delta }})\,|GS{\rangle }_{-})/\sqrt{2}$ which minimizes the energy. The spin fields are consequently well defined. We note some features of the spin field: (i) There is a mirror symmetry about the line *x* = ±*y*. (ii) *σ*_*x*_(**r**) + *σ*_*y*_(**r**) = 0, and *σ*_*x*_(**r**) = *σ*_*y*_(**r**) = 0 along the line *x* = *y*. (iii) ${\sigma }_{z}({\bf{r}})=-\,\frac{{\rm{sgn}}({\rm{\Delta }})}{\pi {\ell }^{2}}{e}^{-2{x}^{2}/{\ell }_{x}}\,\cos \,(4\sqrt{2}{m}^{\ast }xg/\hslash )$ along the line *x* = −*y*, i.e., *σ*_*z*_(**r**) is a spiral. Its period is related to the effective mass and the strength of the SOCs^47^. We find that the exact solution perfectly agrees with the exact diagonalization results shown in Fig. 1. Similar results are found for the case *g*_1_ = −*g*_2_. For large magnetic fields the exact solution for the case without field is no longer a good starting point and the spin texture rotates (See the supplementary material for details).

**Figure 2:**
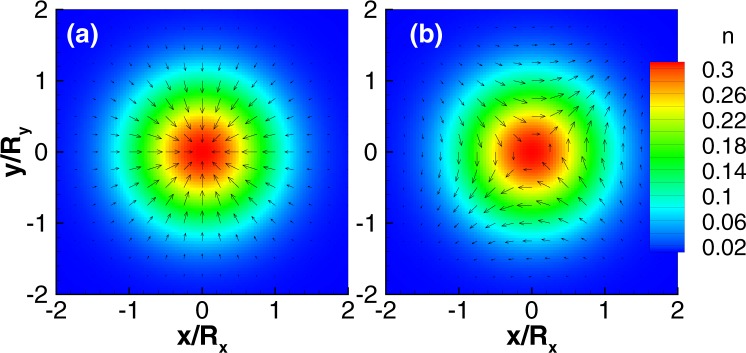
Single-electron QD with *R*_*x*_ = *R*_*y*_ = 15 nm, *B* = 0.1 T (Δ < 0), and (**a**) Rashba SOC $\hslash {g}_{1}=40$ nm · meV only, and (**b**) Dresselhaus SOC $\hslash {g}_{2}=20$ nm · meV only.

Next, we study the case of an isotropic dot in a weak magnetic field with generic strengths of the SOCs *g*_1_ and *g*_2_ based on a standard perturbative calculation. We find that the in-plane spin fields up to first order in *g*_1,2_ are given by

6 $${\sigma }_{x}({\bf{r}})=\xi (r)(r/\ell )({\bar{g}}_{2}\,\sin \,\theta -{\bar{g}}_{1}\,\cos \,\theta ),$$

7 $${\sigma }_{y}({\bf{r}})=\xi (r)(r/\ell )({\bar{g}}_{2}\,\cos \,\theta -{\bar{g}}_{1}\,\sin \,\theta ),$$

and *σ*_*z*_(**r**) = *ξ*(*r*)/2 with $\xi (r)=2{e}^{-{r}^{2}/{\ell }^{2}}/\pi {\ell }^{2}$, *θ* is the polar angle in coordinate space, and the new parameters are

8 $${\bar{g}}_{1,2}=\frac{\hslash {g}_{1,2}}{\ell }\frac{1\pm {\omega }_{c}/(2{\rm{\Omega }})}{\hslash ({\rm{\Omega }}\pm {\omega }_{c}/2)-{\rm{\Delta }}},$$

where we have assumed Δ < 0. The in-plane spin field *σ*(**r**) winds once around the origin and acquires a topological charge *q* = ±1 when ${\bar{g}}_{1}\ne {\bar{g}}_{2}$. If ${\bar{g}}_{1}={\bar{g}}_{2}$, no vortex appears in agreement with the exact solution discussed earlier. If *g*_1_ = 0 or *g*_2_ = 0, *σ*(**r**) obtained perturbatively qualitatively agrees with the numerical solutions shown in Fig. 2, and the vortices even exist in a strong magnetic field beyond the perturbation calculations. We stress that the two vortex configurations are stable and representative for the regime ${g}_{1}\gg {g}_{2}$ and ${g}_{2}\gg {g}_{1}$, respectively (See the supplementary material for details). We further note that under *B* → −*B* the spin field changes direction, *σ*(**r**) → −*σ*(**r**), leaving the topological charge invariant though.

Next, we analyze the rotational symmetry of the two types of SOCs in order to characterize the sign of the winding number. First, we consider the spin field of a dot when only the Rashba SOC is present. The spin field is then invariant under the rotation matrix

9 $${U}_{R}(\vartheta )=(\begin{array}{cc}\cos \,\vartheta  & \sin \,\vartheta \\ -\sin \,\vartheta  & \cos \,\vartheta \end{array}),$$

for $\vartheta \in [0,2\pi ]$, which is rooted in the rotational symmetry of a Rashba dot under the operator ${L}_{z}+\hslash {\sigma }_{z}/2$. Therefore, the in-plane spin rotates clockwise by 2*π* if we move around the center of the dot in a clockwise direction, and hence, its winding number is *q* = +1. On the other hand, the in-plane spin field of a dot with only Dresselhaus SOC being present, is invariant under the action of ${U}_{D}(\vartheta )={U}_{R}(\,-\,\vartheta )$. Along the same line of reasoning, the in-plane spin field then rotates anticlockwise by 2*π* if we move around the center in a clockwise direction. Dresselhaus SOC thus leads to a winding number *q* = −1. In the absence of an external magnetic field *B*, Kramers degeneracy may cancel the spin textures, since there is a global *π* phase difference between the pair. Hence, the vortices should be stabilized by breaking of time-reversal symmetry in which case they are also robust against the ellipticity of the dot (See the supplementary material for details). If the dot is strained, the topological features are not changed, since the spin textures originate from the SOCs of the material.

### Probing the topological state

The total 〈*σ*_*z*_〉 in the presence of SOC is no longer constant as a function of the applied magnetic field and becomes more and more polarized with increasing magnetic field. The distinct behavior of 〈*σ*_*z*_〉 when SOCs are present might be observable experimentally via magnetometry or optically pumped NMR measurements^48–51^.

**Figure 3:**
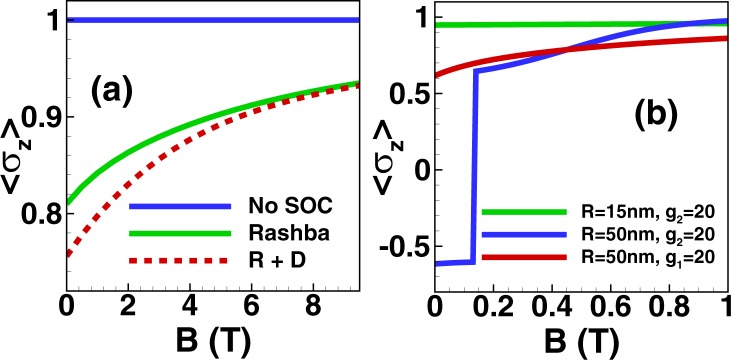
(**a**) 〈*σ*_*z*_〉 in a single-electron InAs dot (*R* = *R*_*x*_ = *R*_*y*_ = 15 nm) without SOC, with Rashba SOC only ($\hslash {g}_{1}=40$ nm · meV), and with both Rashba and Dresselhaus SOCs ($\hslash {g}_{1}=40$ nm · meV, $\hslash {g}_{2}=20$ nm · meV). (**b**) The sign of 〈*σ*_*z*_〉 in a large InAs dot are different for different SOCs. Here *g*_1_ and *g*_2_ are given in units of nm · meV/$\hslash$.

For a small dot with *R* = 15 nm shown in Fig. 3(a), 〈*σ*_*z*_〉 for a Rashba dot and a Dresselhaus dot have the same sign which is opposite to the sign of the Landé factor. However, the measurement of 〈*σ*_*z*_〉 can distinguish the different SOCs if the size of the dot is sufficiently large. To understand this size effect, we again employ perturbation theory and compare the energies of the two states with different spin orientations. For simplicity, we consider an isotropic InAs dot (*ω*_*x*,*y*_ = *ω* and *g*_*L*_ < 0) with only one type of SOC. The sign of 〈*σ*_*z*_〉 is positive in finite magnetic field if there is no SOC. However, if the size is large enough then the sign of 〈*σ*_*z*_〉 can be reversed for a Dresselhaus dot. Suppose that the size of the dot approaches infinity. Then the sign of 〈*σ*_*z*_〉 is reversed if (See the supplementary material for details)

10 $$B < -\,\frac{2\hslash e{m}^{\ast }}{{m}^{\ast }{g}_{L}{\mu }_{B}+\hslash e}\frac{{g}_{2}^{2}}{{g}_{L}{\mu }_{B}}$$

and if the r.h.s. of Eq. (10) is positive. Alternatively, we can also estimate perturbatively the magnitude of the confinement for which the sign is reversed for small magnetic fields leading to the condition $\hslash \omega  < -\,4{g}_{2}^{2}{m}_{e}/{g}_{L}$. For the SOC coupling $\hslash {g}_{2}=20$ meV · nm used in Fig. 3(b) this estimate yields $\hslash \omega  < 1.5$ meV or *R* > 35 nm. This is in reasonable agreement with the numerics where we find a sign reversal for *R* > 49 nm.

Figure 3(b) demonstrates that the sign of 〈*σ*_*z*_〉 for an InAs system with *R* = 50 nm in a weak magnetic field *B* < 0.14 T allows to determine the type of the dominant SOC and thus, indirectly, the topological charge of the dot. We note that for a material with a positive Landé factor, the reversal of 〈*σ*_*z*_〉 will instead occur for a dot with dominant Rashba SOC (See the supplementary material for details). Information stored as topological charge in a quantum dot system can thus be accessed by measuring the sign of 〈*σ*_*z*_〉 in a weak magnetic field.

### The two-electron dot

If there is more than one electron confined in the dot, we need to also consider the Coulomb interaction. The Hamiltonian of the interaction is given by ${H}_{C}=V\,({n}_{1},{n}_{2},{n}_{3},{n}_{4})\,{c}_{{n}_{1}}^{\dagger }{c}_{{n}_{2}}^{\dagger }{c}_{{n}_{3}}{c}_{{n}_{4}}$ where *c* is the electron annihilation operator and *n*_*i*_ = (*n*_*ix*_, *n*_*iy*_, *n*_*s*_) is an index combining the quantum numbers of the two-dimensional oscillator in *x*, *y* direction with the spin index. The interaction matrix elements are given in the Suppl. Mat. The full Hamiltonian with interaction is then *H*_*I*_ = *H* + *H*_*C*_ with *H* as given in Eq. (2). We diagonalize the interacting Hamiltonian exactly to obtain the electron and spin densities. Since the interacting system does contain very rich physics, we restrict the discussion in the following to the case of a dot with two electrons.

**Figure 4:**
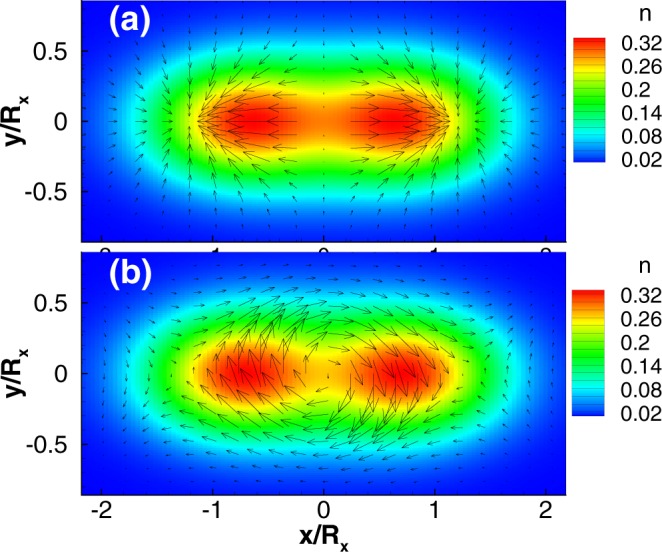
The in-plane spin fields in an elliptic dot with two electrons, *R*_*x*_ = 15 nm, *R*_*y*_ = 10 nm at *B* = 5 T. The colors represent the electron density. (**a**) Rashba SOC only with $\hslash {g}_{1}=40$ nm · meV, and (**b**) Dresselhaus SOC only with $\hslash {g}_{2}=20$ nm · meV.

In a two-electron dot with Coulomb interactions, the spin textures can be much more complex than in the single-electron case. If there is no time reversal symmetry breaking, the texture is cancelled by the Kramers pair. In the presence of a magnetic field, the spin textures appear again with topological charge +1 or −1 if the dot is perfectly isotropic. For an anisotropic quantum dot the electron density will split into two centers in a strong magnetic field even without SOC. With SOCs the spin textures are modified by this density deformation. In the examples shown in Fig. 4, we find in both cases three vortices along the elongated *x* axis. In the Rashba SOC case shown in Fig. 4(a) there are two vortices with *q* = 1 and one with *q* = −1, while there are two vortices with *q* = −1 and one with *q* = 1 in the Dresselhaus SOC case presented in Fig. 4(b). Hence, the total winding numbers are still +1 and −1 in a Rashba SOC and Dresselhaus SOC system, respectively, as in the single-electron dot. Indeed, the spin textures along the edges of the dot are quite similar to the single-particle case. Here interactions are less relevant and the spin textures are thus mainly induced by the SOCs.

**Figure 5:**
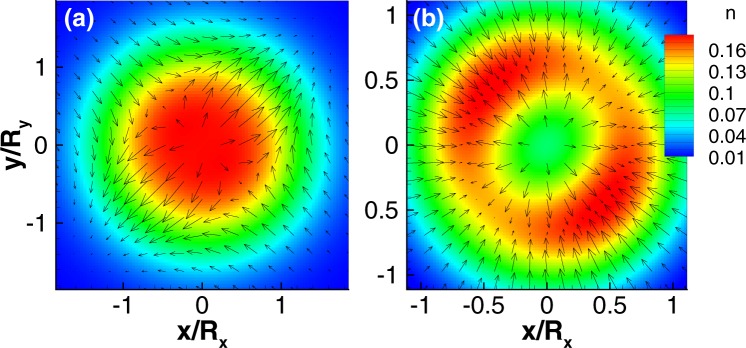
The in-plane spin fields in a two-electron dot with *R*_*x*_ = *R*_*y*_ = 15 nm, and $\hslash {g}_{1}=\hslash {g}_{2}=20$ nm · meV. The colors represent the electron density. (**a**) *B* = 3.5 T, topological charge *q* = −1, and (**b**) *B* = 18 T leading to *q* = +1.

In an isotropic two-electron dot with equal SOCs, *g*_1_ = *g*_2_, we find that both the density profiles and spin textures undergo a dramatic change as a function of the applied magnetic field [Fig. 5]. In this case, the spin and density profiles are determined collectively by *both* the interactions and SOCs. For large magnetic fields we find, in particular, that the electron density splits mirror symmetrically along the line *x* = *y* [Fig. 5(b)], causing also a complete rearrangement of the associated spin texture and a change of the total topological charge. This has to be contrasted with the case of an InAs dot without SOC where the angular momentum of the ground state changes from *L* = −1 to *L* = 3 at about *B* = 17 T leading instead to a ring-shaped electron density. We further note that in a ZnO dot with stronger Coulomb interaction^52^, the splitting of the electron density and the spin textures can be generated in a much lower magnetic field. This splitting—which only occurs if both interactions and SOCs are present—could possibly be observed experimentally and would thus provide an alternative indirect confirmation of a non-trivial spin texture in the dot.

## Conclusions

In summary, the combination of electron confinement and SOCs leads to vortex-like spin textures in the ground state even for a single-electron dot. For dominant Rashba or Dresselhaus SOC, we show the formation of spin vortices. Rashba SOC induces a vortex with topological charge *q* = +1 while the Dresselhaus SOC induces a vortex with *q* = −1. The spin texture can be stabilized by an external magnetic field breaking the time-reversal symmetry and is robust against the strain of the dot. While we have concentrated here on the ground state, we note that non-trivial spin textures can also exist in excited states including states with higher topological charges |*q*| > 1. Contrary to the spin textures in the ground state they are, however, more fragile due to their Kramers partner. Using exact diagonalizations we have shown that these spin vortices do persist also in interacting multi-electron dots. For an elliptic two-electron dot we find, in particular, that more than one spin vortex can exist. In all investigated cases the total topological charge is, however, still *q* = ±1 as in the single-electron case. Physically, this is understood by noting that the spin configuration at the edge of the dot, where the electron density is low, is only weakly affected by the interactions. We thus conjecture that the total topological charge for a spin texture in the ground state of multi-electron dots is always fixed to *q* = ±1. The discussed spin textures in QDs are similar to skyrmions in quantum Hall systems. The locations of the latter are, however, unknown and their existence has so far only been confirmed indirectly by NMR and transport measurements. In contrast, the spin vortices in QD systems are localized at a known position. This might possibly open new avenues for topological spintronics^53–55^ and quantum information applications. Arrays of QDs have, for example, been realized experimentally^56,57^ and have been considered as a potential platform for quantum computation^58–61^. In such an array of QDs with SOCs the ratio of Rashba to Dresselhaus couplings might be tunable by gates over a sufficiently wide range to realize a system with localized and controllable topological charges *q* = ±1. For dominant Rashba or Dresselhaus coupling in a sufficiently large dot, the sign of 〈*σ*_*z*_〉 can be measured to obtain the topological charge. Furthermore, recent progress on measurements of electron wavefunctions in quantum dots by scanning tunneling microscopy^62–64^ might pave the way to a direct observation of the described spin textures by performing spin polarized measurements in the future. QDs might thus provide an easier route to technical applications in the recently emerging field of topological spintronics than the antiferromagnetic heterostructures investigated so far^54,55^ and can potentially give rise to the birth of a novel type of a fully tunable topological system.

## Methods

### Exact and perturbative calculations

For the symmetric dot with equal Rashba and Dresselhaus couplings the Hamiltonian can be rewritten and solved in an oscillator basis by introducing ladder operators. In a weak magnetic field we then obtain the spin textures by a standard first order perturbative calculation in the spin-orbit coupling. Further details are given in the supplemental material.

### Exact diagonalizations

We use our own exact diagonalization code which uses a harmonic oscillator basis which is convenient for the elliptical QDs. For the diagonalization itself standard LAPACK and ARPACK routines are used^65^.

## Electronic supplementary material

Supplemental Material: Unique Spin Vortices and Topological Charges in Quantum Dots with Spin-orbit Couplings
