# Supplementary material for: Unique Spin Vortices and Topological Charges in Quantum Dots with Spin-orbit Couplings
Source: Sci Rep. 2019 Jan 24;9:672. doi: 10.1038/s41598-018-35837-y (PMC6345826; doi:10.1038/s41598-018-35837-y)
Supplement: Supplementary file 1 — Supplemental Material: Unique Spin Vortices and Topological Charges in Quantum Dots with Spin-orbit Couplings [file 41598_2018_35837_MOESM1_ESM.pdf]

Unique Spin Vortices and Topological Charges in Quantum Dots with Spin-orbit Couplings – Supplemental material

Wenchen Luo, Amin Naseri, Jesko Sirker, and Tapash Chakraborty

July 16, 2018

In the supplemental material we present details of the analytical calculations for the single-electron parabolic quantum dot (QD) [1, 2, 3] and additional numerical data demonstrating the stability of the topological spin textures described in the manuscript. In Sec. 1 the derivation of the exact eigenstates for a single-electron dot with equal Rashba and Dresselhaus spin-orbit couplings (SOCs) and without the magnetic field, as well as the lifting of the Kramers degeneracy for small fields are discussed. In Sec. 2 we present the perturbative calculations of the energies required to understand the dependence of the magnetization on the size of the dot. The possibilities of spin reversal are also discussed. In Sec. 3 we present additional numerical data for the single-electron QD showing that (i) for large magnetic fields and $g_1 = g_2$ the spin texture starts to rotate, (ii) the topological charges are robust in the cases $g_1 \gg g_2$ and $g_2 \gg g_1$ and similar to the spin textures with Rashba or Dresselhaus coupling only discussed in the main text, and (iii) the topological charges are also robust against straining of the QD. In Sec. 4 we give explicit formulas for the Coulomb matrix elements for multi-electron QDs.

1 Single-electron dot: Equal Rashba and Dresselhaus couplings

The Hamiltonian of an electron in an isotropic dot $\Omega_{x,y} = \Omega$, $\ell_{x,y} = \ell$ and $g_1 = g_2 = g$ without magnetic field is equivalent to a two-component quantum Rabi model. In this case the Hamiltonian (2) in the main text reads,

$$H = \frac{\mathbf{p}^2}{2m^*} + \frac{1}{2}m^*\omega^2\left(x^2+y^2\right) - \left(p_x - p_y\right) g \left(\sigma_x + \sigma_y\right). \tag{S.1}$$

We now define the ladder operators of the quantum harmonic oscillator

$$a_\mu = \sqrt{\frac{m^*\omega}{2\hbar}}\left(\mu + \frac{i}{m^*\omega}p_\mu\right), \tag{S.2}$$

and use $a_x = -ib_x, a_y = ib_y$, to transform the Hamiltonian to

$$H = b_x^\dagger b_x + b_y^\dagger b_y + G\left(b_x^\dagger + b_x + b_y^\dagger + b_y\right)\frac{1}{\sqrt{2}}\left(\sigma_x + \sigma_y\right), \tag{S.3}$$

where $G = g\sqrt{\hbar m^*\omega}$. Then, performing a unitary transformation

$$U = \frac{1}{\sqrt{2}}\begin{pmatrix} 1 & e^{-i\pi/4} \\ e^{i\pi/4} & -1 \end{pmatrix}, \tag{S.4}$$

leads to

$$UHU^{\dagger} \;=\; b_x^{\dagger}b_x + b_y^{\dagger}b_y + G\left(b_x^{\dagger} + b_x + b_y^{\dagger} + b_y\right)\sigma_z, \tag{S.5}$$

which is a two-component quantum Rabi model with zero splitting [4]. The case of $g_1 = -g_2$ can be solved in a similar manner.

In the main text, we show in Eq. (5) that the ground states are a degenerate Kramers pair. If the magnetic field is infinitesimal then the degeneracy is lifted. Since $_{\pm}\langle GS|\, L_z\, |GS\rangle_{\pm} = 0$, the unique ground state can be found to lowest order by minimizing the Zeeman energy only. We use the ansatz

$$|GS\rangle = A\,|GS\rangle_{+} + B\,|GS\rangle_{-}\,. \tag{S.6}$$

with coefficients A, B. The Zeeman energy is then proportional to

$$\begin{aligned}\langle GS|\, \Delta\sigma_z\, |GS\rangle \;&=\; -\Delta\int_{-\infty}^{\infty} dxdy\left(AB^{*}e^{-i2\sqrt{2}g\ell\frac{m^{*}}{\hbar}(x-y)} + A^{*}Be^{i2\sqrt{2}g\ell\frac{m^{*}}{\hbar}(x-y)}\right)e^{-x^2-y^2} \\ &=\; -\Delta\left(AB^{*} + A^{*}B\right)\pi e^{-4\left(g\ell\frac{m^{*}}{\hbar}\right)^2}. \end{aligned}\tag{S.7}$$

For $\Delta > 0$ the minimization of the Zeeman energy requires $A = B = \frac{1}{\sqrt{2}}$ while it requires $A = -B = \frac{1}{\sqrt{2}}$ for $\Delta < 0$. Hence,

$$|GS\rangle = \frac{|GS\rangle_{+} + \mathrm{sgn}\,(\Delta)\,|GS\rangle_{-}}{\sqrt{2}}. \tag{S.8}$$

The other calculations presented in the main text are the standard first-order perturbative calculations.

2 Single-electron dot: size effects

For a small dot (e.g. the case $R_x = R_y = 15$nm for an InAs dot), the sign of $\langle\sigma_z\rangle$ in the ground state cannot be changed by the SOCs. However, if the size is enlarged, then the sign of $\langle\sigma_z\rangle$ may be changed due to SOCs. We compute the perturbative energies for different spin projections. The first-order correction of the energy is zero, so we consider the second-order corrections,

$$E_{\pm} = E_{\pm}^{0} + E_{\pm}^{(2)} = \sum_k \frac{\left|\langle k^{(0)}|\, H_{SOC}\, |n^{(0)}\rangle\right|^2}{E_n^{(0)} - E_k^{(0)}}.$$

If there is no magnetic field, both states are still degenerate due to time reversal symmetry, $E_+ = E_-$. In general,

$$E_{+} \;=\; \frac{\Delta}{2} + \left(\frac{\hbar g_2}{\ell}\right)^2 \frac{\left(1 - \frac{\omega_c}{2\Omega}\right)^2}{\Delta - \hbar\left(\Omega - \frac{\omega_c}{2}\right)} + \left(\frac{\hbar g_1}{\ell}\right)^2 \frac{\left(1 + \frac{\omega_c}{2\Omega}\right)^2}{\Delta - \hbar\left(\Omega + \frac{\omega_c}{2}\right)}, \tag{S.9}$$

$$E_{-} \;=\; -\frac{\Delta}{2} + \left(\frac{\hbar g_1}{\ell}\right)^2 \frac{\left(1 - \frac{\omega_c}{2\Omega}\right)^2}{-\Delta - \hbar\left(\Omega - \frac{\omega_c}{2}\right)} + \left(\frac{\hbar g_2}{\ell}\right)^2 \frac{\left(1 + \frac{\omega_c}{2\Omega}\right)^2}{-\Delta - \hbar\left(\Omega + \frac{\omega_c}{2}\right)}, \tag{S.10}$$

where E_+ is for the state with dominant spin $|+\rangle$ while E_- is for dominant spin $|-\rangle$. We have to compare these two energies to figure out the true ground state. If there is only Rashba

coupling present, then

$$E_{R+} = \frac{\Delta}{2} + \left(\frac{\hbar g_1}{\ell}\right)^2 \frac{\left(1+\frac{\omega_c}{2\Omega}\right)^2}{\Delta - \hbar\left(\Omega + \frac{\omega_c}{2}\right)}, \tag{S.11}$$

$$E_{R-} = -\frac{\Delta}{2} + \left(\frac{\hbar g_1}{\ell}\right)^2 \frac{\left(1-\frac{\omega_c}{2\Omega}\right)^2}{-\Delta - \hbar\left(\Omega - \frac{\omega_c}{2}\right)}. \tag{S.12}$$

If, on the other hand, there is only Dresselhaus present, then

$$E_{D+} = \frac{\Delta}{2} + \left(\frac{\hbar g_2}{\ell}\right)^2 \frac{\left(1-\frac{\omega_c}{2\Omega}\right)^2}{\Delta - \hbar\left(\Omega - \frac{\omega_c}{2}\right)}, \tag{S.13}$$

$$E_{D-} = -\frac{\Delta}{2} + \left(\frac{\hbar g_2}{\ell}\right)^2 \frac{\left(1+\frac{\omega_c}{2\Omega}\right)^2}{-\Delta - \hbar\left(\Omega + \frac{\omega_c}{2}\right)}. \tag{S.14}$$

We note that the perturbation theory becomes invalid if the denominators in the perturbative corrections are close to zero. In most cases, however, the perturbation theory works fine, especially if the magnetic field is not too large.

If the dot is very large (approaching infinity) then $\Omega \to \omega_c/2$ and

$$E_{D+} - E_{D-} = g_L \mu_B B + \frac{2\hbar e}{g_L \mu_B + \frac{\hbar e}{m^*}} g_2^2, \tag{S.15}$$

$$E_{R+} - E_{R-} = g_L \mu_B B + \frac{2\hbar e}{g_L \mu_B - \frac{\hbar e}{m^*}} g_1^2. \tag{S.16}$$

Let us consider, in particular, an InAs dot. If only Rashba SOC is present, then the ground state is always the state with energy E_{R+} ($E_{R+} - E_{R-} < 0$ because $g_L < 0$). Therefore $\langle \sigma_z \rangle > 0$ and $\langle \sigma_z \rangle \to 1$ when the magnetic field is strong. However, when only the Dresselhaus SOC is present, the sign of $\langle \sigma_z \rangle$ can be reversed. This happens because for weak magnetic field the ground state can change to the state with energy E_{D-} ($E_{D+} - E_{D-} > 0$ leads to Eq. (10) in the manuscript) with $\langle \sigma_z \rangle < 0$.

Consider, on the other hand, a material with positive Landé factor, $g_L > 0$, for example ZnO. If only Dresselhaus SOC is present, then the ground state is the state with energy E_{D-}, since $E_{D+} - E_{D-} > 0$. Therefore we always have $\langle \sigma_z \rangle < 0$. When only the Rashba SOC exists, then the sign of $\langle \sigma_z \rangle$ can be reversed. With regard to a possible sign change of $\langle \sigma_z \rangle$ in the ground state of the dot the roles of Dresselhaus and Rashba SOC are thus reversed if the sign of the Landé factor g_L is changed. Note that in both cases (for the $g_L < 0$ Dresselhaus dot and for the $g_L > 0$ Rashba dot), the effective mass m^* should not be too large, $m^* < 2m_e/|g_L|$, otherwise a sign reversal is not possible.

In Fig. S1, we compare the spin textures in InAs dots with different sizes (a) $R = 15$nm and (b) $R = 50$nm. The Dresselhaus SOC is the same in both cases, $\hbar g_2 = 20$ nm· meV, and the Rashba SOC is zero. We find that the topological charge is the same while σ_z is reversed by the size effect.

In other materials we can do the same analysis, i.e. compare the energies between E_{R+} and E_{R-} or between E_{D+} and E_{D-} to find if there is a sign change of $\langle \sigma_z \rangle$. The perturbative energies may not be very accurate for a large dot in strong magnetic fields, but the numerical calculations directly yield reliable results for $\langle \sigma_z \rangle$.

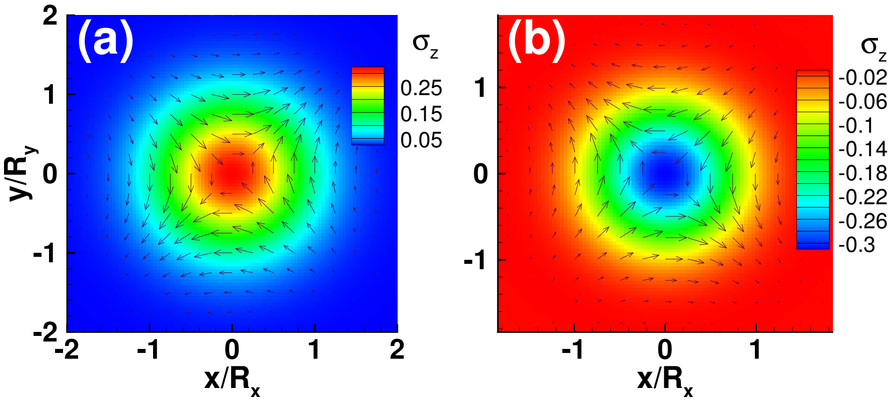

Figure S1: (Color online) The spin textures in InAs dots with Dresselhaus SOC $\hbar g_2 = 20$ nm·meV. The sizes are (a) $R = 15$nm and (b) $R = 50$nm.

3 Single-electron dot: Numerical results

In Fig. 1(a) of the main text we have shown that the in-plane spin texture in a weak magnetic field for the case $g_1 = g_2$ is mirror symmetric around the line $x = y$, consistent with the perturbative calculation. For larger fields the perturbative ground state given above is, however, no longer a good starting point. In Fig. S2, we show how the in-plane spin textures evolve with increasing magnetic field. All these results show a mirror symmetry about the line $x = \pm y$. However, when the magnetic field becomes stronger the spins start to rotate leading to a spin texture similar to the case of Rashba SOC only. Note that the in-plane spin components are weaker than in the Rashba case though because the spin becomes more and more polarized along the z-direction.

In Fig. 2 of the main text we have shown the spin vortices in a single-electron dot if only the Rashba or the Dresselhaus SOC is present. In Fig. S3 we show that these results are indeed representative for the regimes $g_1 \gg g_2$ and $g_2 \gg g_1$. We also note that even for larger magnetic fields the topological properties are not changed, although the spin textures are weakened. States with higher topological charge $|q| > 1$ may exist in the excited states. Contrary to the spin textures in the ground state they are, however, fragile due to their Kramers partner.

Finally, we also show that the spin texture is robust against the eccentricity of the dot. We consider an elliptical InAs dot with $R_x = 15$nm and $R_y = 10$nm. From Fig. S4 it is obvious that the distortion of the dot does not qualitatively change the structure of the spin vortex.

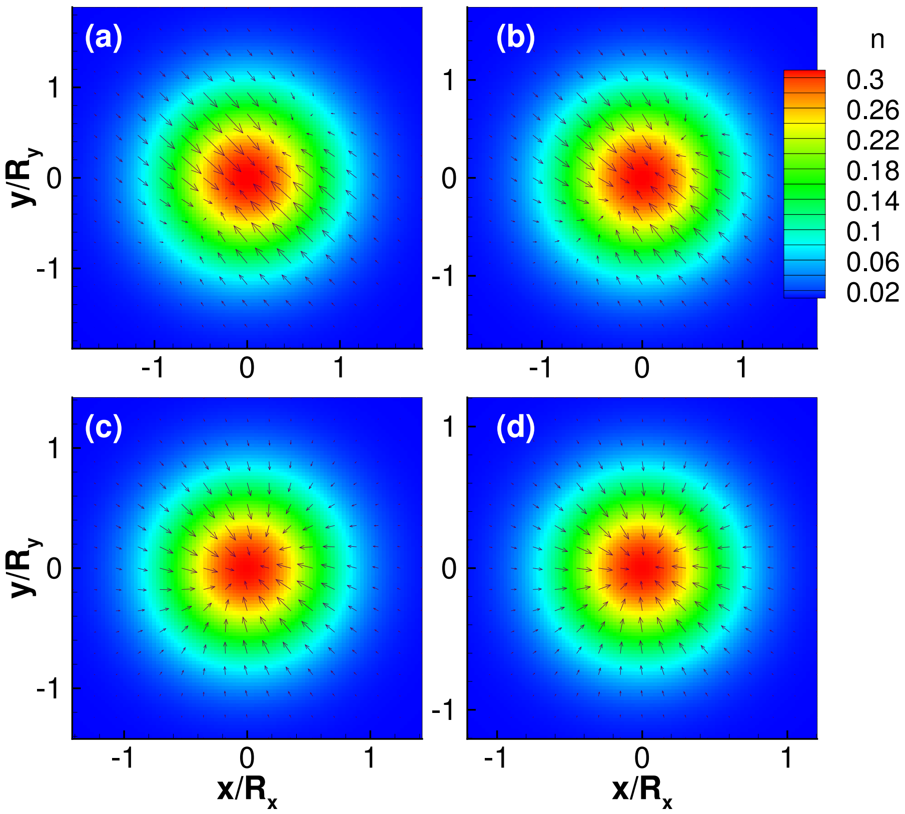

Figure S2: (Color online) The in-plane spin field with $\hbar g_1 = \hbar g_2 = 20$ nm$\cdot$ meV in a single-electron dot with $R_x = R_y = 15$nm at magnetic fields (a) $B = 3$T, (b) $B = 5$T, (c) $B = 10$T, and (d) $B = 15$T.

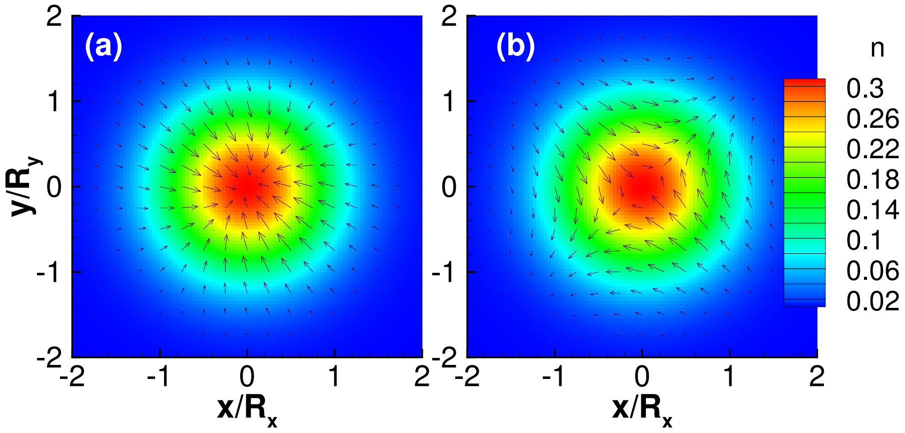

Figure S3: (Color online) The in-plane spin field in an isotropic single-electron dot with $R_x = R_y = 15$nm at $B = 0.1$T. The SOCs are (a) $\hbar g_1 = 20$ nm$\cdot$ meV and $\hbar g_2 = 5$nm$\cdot$ meV, and (b) $\hbar g_1 = 5$nm$\cdot$ meV and $\hbar g_2 = 20$nm$\cdot$meV.

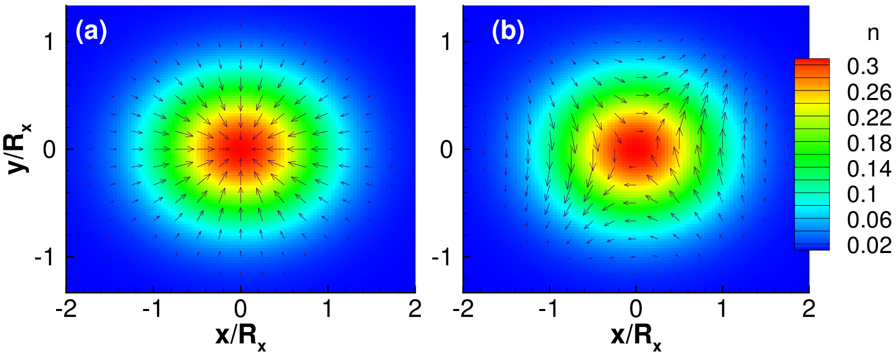

Figure S4: (Color online) The in-plane spin field in a single-electron dot with $R_x = 15$nm, $R_y = 10$nm in $B = 0.1$T. The SOCs are (a) $\hbar g_1 = 20$ nm· meV and $\hbar g_2 = 0$, and (b) $\hbar g_1 = 0$ and $\hbar g_2 = 20$nm·meV.

4 Multi-electron dots: Coulomb interaction matrix elements

Below we explicitly display the Coulomb interaction matrix elements [3] used in the exact diagonalization method for multi-electron dots

$$\begin{aligned} V(n_1, n_2, n_3, n_4) &= \frac{2}{\pi} \frac{e^2}{\epsilon\sqrt{\ell_x \ell_y}} \phi(n_{1x}, n_{4x})\, \phi(n_{1y}, n_{4y})\, \phi(n_{2x}, n_{3x})\, \phi(n_{2y}, n_{3y}) \\ &\quad (-1)^{|n_{2x}-n_{3x}|+|n_{2y}-n_{3y}|}\, i^{|n_{1x}-n_{4x}|+|n_{1y}-n_{4y}|+|n_{2x}-n_{3x}|+|n_{2y}-n_{3y}|} \\ &\quad \int_0^\infty dx dy\, \Phi(n_{1x}, n_{4x}, x)\, \Phi(n_{2x}, n_{3x}, x) \frac{\Phi(n_{1y}, n_{4y}, y)\, \Phi(n_{2y}, n_{3y}, y)}{\sqrt{\frac{\ell_y}{\ell_x}x^2 + \frac{\ell_x}{\ell_y}y^2}}. \end{aligned} \quad (S.17)$$

Here ϵ is the dielectric constant and

$$\begin{aligned} \phi(n, m) &= \sqrt{\frac{2^{\min(n,m)} \min(n, m)!}{2^{\max(n,m)} \max(n, m)!}}, \\ \Phi(n, m, x) &= x^{|n-m|} e^{-\frac{1}{4}x^2} L^{|n-m|}_{\min(n,m)}\left(\frac{x^2}{2}\right) \end{aligned}$$

with the Laguerre polynomial L.

References

[1] V. Fock, Z. Phys. **47**, 446 (1928).

[2] C.G. Darwin, Proc. Cambridge Philos. Soc. **27**, 86 (1930).

[3] T. Chakraborty, *Quantum Dots* (Elsevier, Amsterdam 1999).

[4] I.I. Rabi, Phys. Rev. **49** 324 (1936); I.I. Rabi, Phys. Rev. **51** 652 (1937); D. Braak Phys. Rev. Lett. **107** 100401 (2011); Qing-Hu Chen, Chen Wang, Shu He, Tao Liu, and Ke-Lin Wang, Phys. Rev. A **86**, 023822 (2012).
